# Creep behavior of clayey soil and its model prediction in the Cangzhou land subsidence area

**DOI:** 10.1038/s41598-025-93928-z

**Published:** 2025-03-17

**Authors:** Jianfeng Qi, Yongjie Xie, Chen Li, Haipeng Guo, Yunlong Wang

**Affiliations:** 1 Hebei Cangzhou Groundwater and Land Subsidence National Observation and Research Station, Cangzhou, 061000 China; 2 Key Laboratory of Intelligent Detection and Equipment for Underground Space of Beijing-Tianjin-Hebei Urban Agglomeration, Ministry of Natural Resources, Hebei GEO University, Shijiazhuang, 050031 China; 3 China Institute of Geo-Environment Monitoring, Beijing, 100081 China

**Keywords:** Creep behavior, Clayey soil, Land subsidence, Nonlinear creep model, Natural hazards, Engineering

## Abstract

In the Cangzhou area of China, groundwater over-exploitation has led to serious land subsidence, and the creep deformation of aquitards has been monitored and found to be closely related to the development of land subsidence. The objective of this paper is to develop a computational model to reflect the creep deformation of aquitards in this area. Firstly, creep tests were conducted on clayey soils with burial depths ranging from 65.7 to 121.7 m. The results show that the total strain consists of three parts: instantaneous strain, primary consolidation strain and creep strain. Creep-time curves and isochronous creep stress–strain curves under stepwise loading were obtained by using the Boltzmann superposition principle, and both types of curves were characterized by nonlinearity, and the creep curves as a whole showed a trend of stable development. Secondly, on the basis of analyzing the advantages and disadvantages of the classical rheological models for clayey soils, a nonlinear creep model of NCE_CS that can take into account the influence of primary consolidation is proposed. The model contains five parameters, which can be solved by using genetic algorithm, and then a simple determination method of the parameters is proposed. Finally, by comparing with the test data and the calculation results of four classical creep models, it is confirmed that the NCE_CS model can fit the creep curves better. The NCE_CS model was also successfully used to estimate the creep behavior in another subsidence area located in Renqiu City in northwest of Cangzhou. This study will provide a basis for quantitative calculation of creep of clayey soils in the Cangzhou area.

## Introduction

The Cangzhou Plain is an extremely water-deficient area under the dual pressure of population and environment. Because of the scarcity of surface water, the entire region relies mainly on over-exploitation of groundwater to sustain the growing needs of national economic development. Land subsidence poses a threat to the normal operation of municipal facilities such as urban water supply and gas supply, leading to a decrease in the city’s flood control capacity. Land subsidence is mainly due to the compression deformation of soil layers caused by over-exploitation of groundwater, with the compression deformation of clayey soil layers contributing the most to land subsidence. Surface water accounts for about 20% of the total water use in Cangzhou, and the remaining 80% is groundwater^1^. With the increase in groundwater extraction, the groundwater level declined and a regional groundwater level depression cone was gradually formed. As a result, the pore water pressure in the aquifer decreased and the clayey soil layers lost water due to compaction, leading to land subsidence. The most severe land subsidence occurred in 2017, with 1400 km^2^ of land experiencing annual subsidence rates exceeding 50 mm^2^.

To mitigate land subsidence, local government had implemented policies since 2005 to limit the extraction of groundwater and to increase the supply of surface water from the Dalangdian Reservoir and the South-to-North Water Diversion Project. As a result, the groundwater level of Cangzhou City has risen significantly, and the land subsidence in the urban area of Cangzhou City has been controlled to a certain extent, but the land subsidence is still in a relatively rapid development stage^2,3^. At present, the severe subsidence area in Cangzhou covers an area of about 300 km^2^, mainly distributed in the west and south of Cangzhou. In the central urban area, the maximum cumulative subsidence has surpassed 2.5 m^1^.

**Fig. 1:**
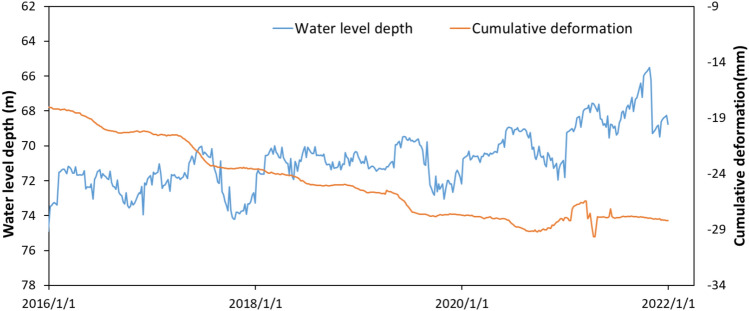
Time series of groundwater level depth and cumulative deformation in a confined aquifer in Cangzhou.

The relationship between the change of groundwater level and land subsidence in Cangzhou City is shown in Fig. 1. The data were obtained from highly sensitive borehole extensometers provided by the Hebei Cangzhou Groundwater and Land Subsidence National Observation and Research Station^2^.The groundwater level fluctuates and rises after November 2019, but the compression deformation of the aquifer continues to increase, indicating that there is delayed creep in clayey soils. Based on the data of borehole extensometers and groundwater level monitoring, some researchers have suggested the presence of inelastic creep in clayey soils^4–6^. Although the creep deformation of the aquitard is relatively small compared with the primary consolidation deformation, the land subsidence caused by creep deformation still needs attention due to the large cumulative thickness of aquitards in Cangzhou. Therefore, it is of great significance to analyze the creep characteristics of clayey soil and establish the corresponding creep model for the prevention and control of land subsidence.

The macroscopic mechanical behavior of clay is usually represented by constitutive models. Commonly used constitutive models include elastoplastic constitutive models, critical state constitutive models and creep constitutive models^7–9^. At present, various creep models of soft soil have been developed, including empirical, semi-empirical and semi-theoretical and theoretical models. The theoretical models were generally obtained through rigorous mechanical analysis and derivation, combined with the verification of the results of creep tests. Yuan et al. proposed a generalized elastoplastic-visco model, MIT-SR, which is capable of describing the time-dependent characteristics of creep to shear behavior in clays^10^. Ding et al. introduced a constitutive model that can reproduce the shear creep characteristics under complex stress conditions, and the predicted curves of the model show good agreement with the experimental data^11^. Chen et al. suggested a constitutive model to describe the time-dependent stress–strain behavior of clays at different temperatures, where the viscoplastic strain rate of the soil can be described using three state variables: effective stress, strain, and temperature^12^. Such models were rarely applied in practical engineering due to the difficulty in solving them.

Empirical models are widely used in practical engineering applications due to the simplicity of expression, easy access to parameters, etc. Singh and Mitchell proposed an exponential function to describe the stress–strain relationship and a power function to describe the strain–time relationship, on the basis of which a most commonly used three-parameter creep model was developed^13^. Subsequently, the researchers proposed some improved empirical creep models and shear creep theories based on creep test results^14–20^. Such models usually use hyperbolic functions and power functions to describe the stress–strain relationship and strain–time relationship. Semi-empirical and semi-theoretical models usually combine the creep empirical relation with mechanical theory to describe the creep behavior of soft soil. Liu et al. established a four-element fractional-order creep model for soft clay based on the fractional-order Burgers creep model, which can more accurately describe the creep curves of soft clay compared to the Burgers model^21^. Chen et al. proposed a four-parameter hyperbolic equation for the relationship between creep volumetric strain, stress, and time, and then a new two-parameter hyperbolic equation for creep was proposed by introducing a reference time, and a five-parameter soft clay creep model was further developed^22,23^. The mechanism of such models is clear, but the model parameters are more numerous and their physical significance is unclear or their values are difficult to determine.

Most of the rheological element models belong to the semi-empirical-semi-theoretical models, which have received attention from researchers for describing the complex mechanical behavior of clayey soils using combinations of basic components. Based on the experimental results, many researchers have analyzed stress–strain curves and stress–strain–time curves and proposed improved nonlinear creep constitutive models using combinations of basic elements^24–30^. Classical rheological element models include Kelvin model, Maxwell model, Burgers model, etc. As the study continued, the researcher found that the creep properties of some clayey soils could not be described by the above elemental models. The classical element models were improved by connecting the elements in series and parallel to describe the creep properties of clayey soils. For example, the models were improved by introducing nonlinear Hooke element and nonlinear dashpot element^24,25^. In recent years, in order to describe the complex creep behaviors of soft clay such as steady-state creep, unsteady-state creep and accelerated creep, the fractional-order nonlinear element had been introduced into the classical rheological element models, whose calculation results were in good agreement with the experimental data^30,31^. In addition, numerical and analytical methods were utilized to solve the established constitutive models^32,33^, which provided a reference for the calculation and analysis of deformation of multi-layer clay soil. In general, the more creep properties are reflected by a creep model, the more parameters the model needs, which often cannot be directly determined by conventional laboratory or field tests. In order to reduce the testing cost and improve the accuracy of model parameter identification, some evolutionary algorithms have been applied in model parameter identification and optimization and have achieved good results^34–39^.

The research objective of this paper is to develop a nonlinear model reflecting the creep properties of deep clayey soils. Firstly, creep tests were carried out on clayey soils in aquitards in a typical subsidence zone in Cangzhou. Secondly, on the basis of analyzing the creep and deformation properties of clayey soils, a nonlinear creep model of NCE_CS is proposed by improving the Kelvin body in the Merchant model and replacing the elastic element by the power function element, and optimizing and solving the model parameters using genetic algorithm. This work will provide a theoretical basis for the quantitative calculation of creep deformation of aquitards in Cangzhou area.

## Materials and methods

### Study area

**Fig. 2:**
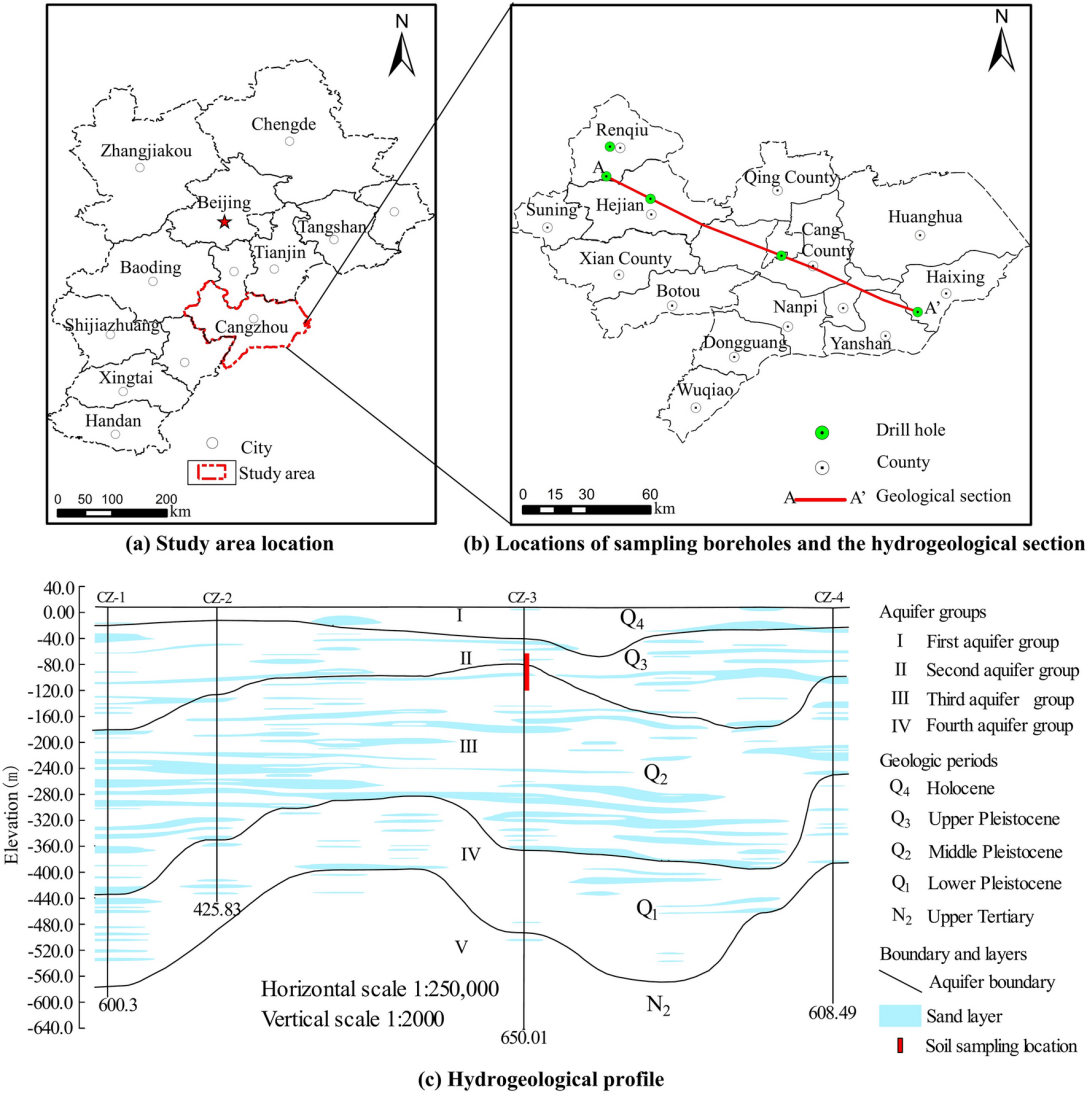
Study area and the location of sampling boreholes, and a typical hydrogeological cross section A–A′.

Cangzhou City is located in the eastern part of the North China Plain (Fig. 2a) and has a flat topography. The predominant stratigraphy in this area is loose unconsolidated Quaternary sediments with the thickness from 200 m to over 600 m (Fig. 2b, c), consisting primarily of sand, silt, and clay. The predominant type of groundwater is pore water in the loose Quaternary sediments, and the aquifer systems typically have multiple sandy aquifers separated by extensive and thick silt or clay aquitards (Fig. 2c). From top to bottom, there are mainly four aquifer groups, which roughly correspond to the Holocene (Q_4_), the Upper Pleistocene (Q_3_), the Middle Pleistocene (Q_2_) and the Lower Pleistocene (Q_1_), respectively. The bottom depths of the first, second, third and fourth aquifer groups are 40~60 m, 120~170 m, 250~350 m, and 350~550 m, respectively. Groundwater in the first and the second aquifer groups has unconfined or semi-confined hydraulic characteristics, and groundwater in the third and the fourth aquifer groups is confined.

### Geotechnical testing

The thickness of the Quaternary clay layer in Cangzhou City is large, reaching a thickness of nearly 200 m. Since the shallow groundwater in Cangzhou is dominated by saline/brackish water, the aquifer groups below the first aquifer group are the main groundwater abstraction layers, and their compression is also the reason for the development of land subsidence. In this paper, four undisturbed clayey soil samples below 65.7 m were selected for the geotechnical creep tests, which were taken from the urban area of Cangzhou, and the sampling location is shown in Fig. 2c.

**Fig. 3:**
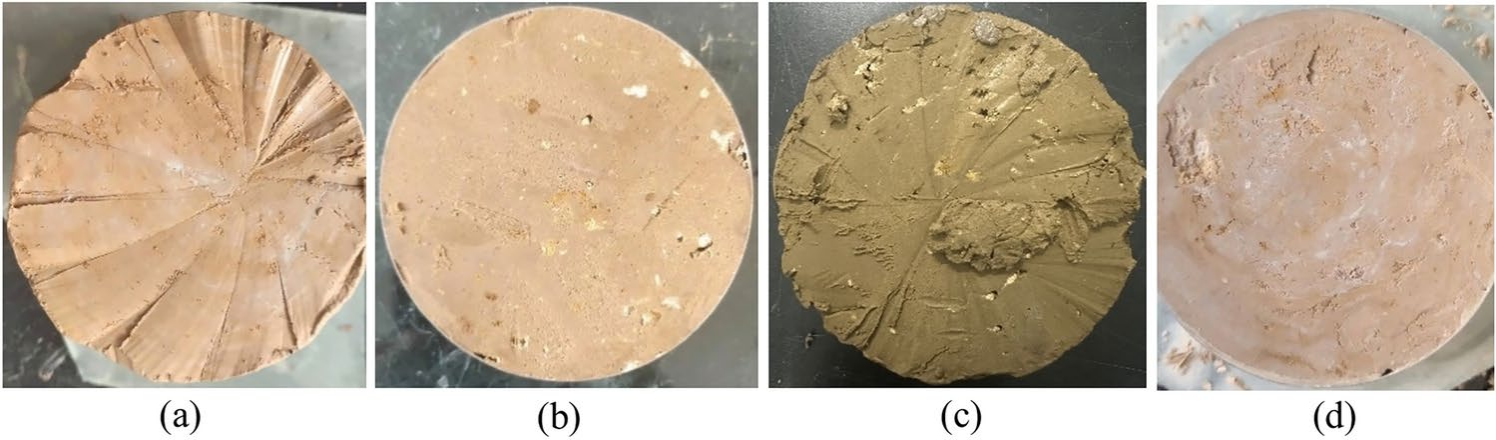
Samples of undisturbed soil used in the tests. (**a**) N1 (**b**) N2 (**c**) N3 (**d**) N4.

The characteristics of the four undisturbed samples (Fig. 3) are as follows: (1) The clay is mainly yellowish-brown and yellow–brown in color, and its composition is nonuniform, containing small sand grains and white calcium sediments; (2) The liquid index is less than 0.25, and it is in the state of hard-plasticity or hardness. The basic physical parameters are shown in Table 1.

**Table 1:** Basic physical properties of undisturbed soil samples N1–N4.

Soil sample number	Depth (m)	Water content *w* (%)	Specific gravity *G*_s_	Void ratio *e*	Liquid limit *W*_L_ (%)	Plastic limit *W*_P_ (%)	Plasticity index *I*_P_	Saturation *S*_r_ (%)	Gravity stress (kPa)
N1	− 65.7	24.85	2.72	0.724	51.1	22.48	28.62	93	867.71
N2	− 96.2	15.55	2.72	0.462	51.75	12.07	39.68	92	1185.99
N3	− 101.3	19.42	2.72	0.562	43.74	12.44	31.4	94	1240.74
N4	− 121.7	22.87	2.72	0.638	39.65	24.29	15.36	97	1460.94

**Fig. 4:**
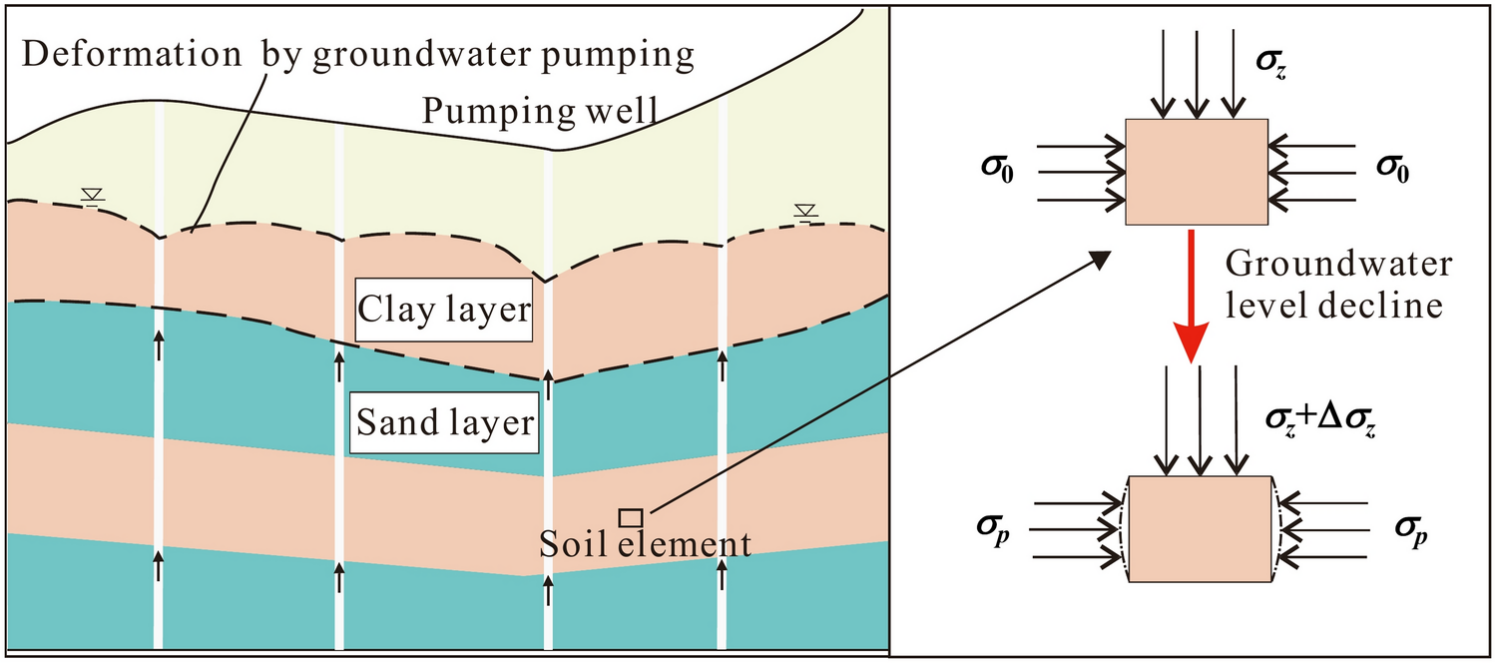
Schematic diagram of the stress state change in the soil due to groundwater level decline.

Under groundwater abstraction conditions, the groundwater level continues to fall, forming multiple localized cones of groundwater depression, which gradually connect to form a regional depression cone. The groundwater level depth in the center of the depression cone in Cangzhou area has exceeded 90 m. The changes in the stress states of the deep clayey soil due to the drop of groundwater level are shown in Fig. 4.

For the deep clayey soil units, the additional stress caused by drop in groundwater level can be expressed as^40^

1 $$\Delta \sigma_{z} \, = \, (\gamma_{sat} - \gamma^{\prime } )\Delta h = \gamma_{w} \Delta h$$

where Δ*σ*_*z*_ is the vertical additional stress; *γ*_sa*t*_, *γ*′ and *γ*_w_ are the saturated unit weight and buoyant unit weight of soil and unit weight of water, respectively; Δ*h* is the value of groundwater level decline.

Clayey soil units will have a tendency to deform laterally when subjected to vertical additional stress Δ*σ*_*z*_, at which point the horizontal pressure changes from static earth pressure *σ*_0_ to passive earth pressure *σ*_*p*_. According to Rankine’s earth pressure theory (Eq. 2), the passive earth pressure can be expressed as^40^

2 $$\sigma_{p} = \gamma zk_{p} + 2c\sqrt {k_{p} } ,\;k_{p} = \tan^{2} \left( {45 + \frac{\phi }{2}} \right)$$

where, *γ* , *γ*_sa*t*_ and *γ*′ are the natural unit weight, saturated unit weight and buoyant unit weight of soil, respectively; *k*_p_ is the passive earth pressure coefficient, greater than 1; *z* is the depth of the soil element; *c* and *ϕ* are the cohesive force and internal friction angle, respectively.

When the depth z is large, the additional stresses Δ*σ*_*z*_ induced by the drop of groundwater level are much smaller than *σ*_*p*_, so the lateral deformation can be neglected. In this way, it is reasonable to use one-dimensional compression test to study the creep behavior of deep clayey soil.

The one-dimensional creep tests were carried out with reference to the Standard for Geotechnical Test Methods (China Planning Press, 2019), and the test apparatus was a WG-type high-pressure consolidation apparatus produced by Nanjing Soil Instrument Factory. The specimen was 2.0 cm high and had a cross-sectional area of 30.0 cm^2^, and the specimen was drained on both sides during the consolidation process. The specimens were loaded in a stepwise loading mode, and the loading scheme of specimens N1, N2 and N4 was as follows: 100 kPa → 200 kPa → 400 kPa → 800 kPa → 1600 kPa → 3200 kPa, and the loading time for each level was 4d, which lasted for a total of 24d. The loading scheme of specimen N3 was as follows: 100 kPa → 400 kPa → 800 kPa → 1600 kPa → 3200 kPa, and the loading time for each level was 8d, which lasted for 40d in total. The laboratory temperature was controlled at 24 ± 1 °C during the test to ensure that the outside temperature had minimal effect on the test results.

## Creep behavior of the clayey soils

### Analysis of creep curves

**Fig. 5:**
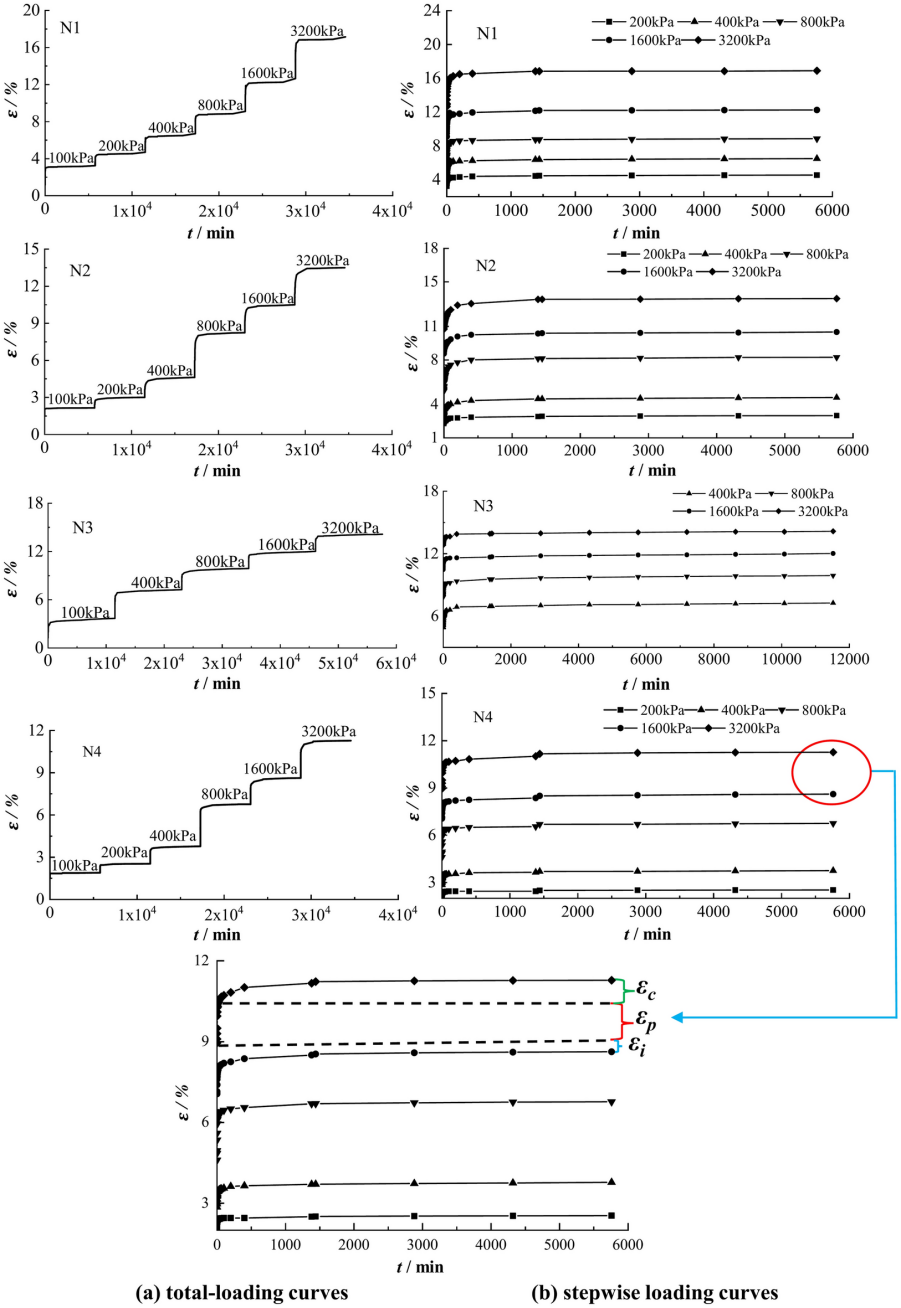
Creep testing curves of the clayey soil samples N1–N4.

Figure 5 shows the strain–time curves of N1~N4 specimens under each level of load. As shown in Fig. 5a, the strain–time curves under the whole loading present a stepped shape, which has an obvious turning point at the initial stage with the increase of each loading, and then enters the steady-state creep stage. The grading loading testing (Fig. 5b) shows that the total strain $\varepsilon$ of each loading is composed of three parts: instantaneous strain $\varepsilon_{i}$, primary consolidation strain ${\varepsilon }_{p}$ and creep strain $\varepsilon_{c}$ (Eq. 3):

3 $$\varepsilon = \varepsilon_{i} + \varepsilon_{p} + \varepsilon_{c}$$

The reasons for the turning point of the strain–time curve are as follows. The specimen generates excess pore water pressure under loading, and the effective stress increases during the gradual dissipation of pore pressure, leading to the primary consolidation deformation which accounts for a large proportion of the total deformation. After the pore water is completely discharged, the deformation is mainly dominated by the creep of soil particles and cohesive water, accounting for a small proportion of the total deformation. According to Casagrande’s method, *e*-log(*t*) curves were made to divide the primary and secondary consolidation. Casagrande believed that the end time of primary consolidation in clayey soils is about 200 min. The test results for specimens N1~N4 showed that the end time of primary consolidation was about 400 min, and thus 400 min was chosen as the time of creep occurrence.

**Fig. 6:**
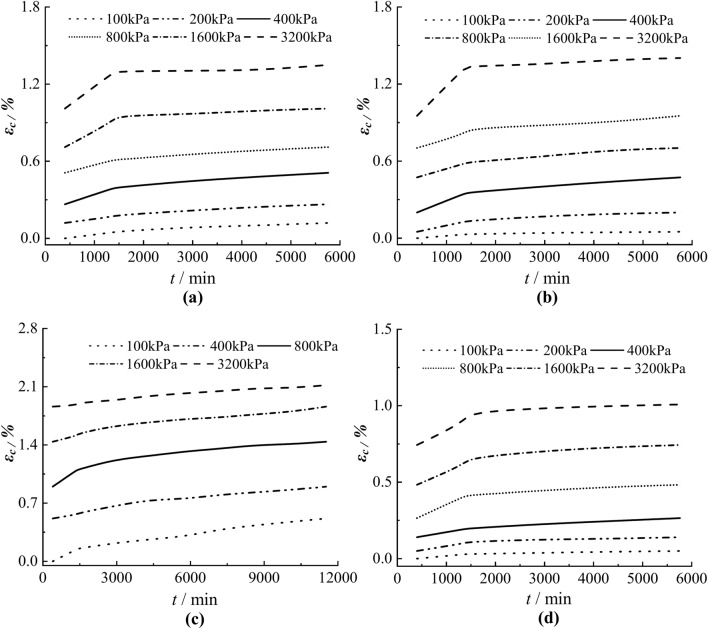
*ε*_*c*_-*t* relations in the creep stage under grading loading. (**a**) N1 (**b**) N2 (**c**) N3 (**d**) N4.

For specimens N1~N4, because the duration of stepwise loading is the same and the deformation is in a steady development trend, so it is more appropriate to use the Boltzmann superposition principle to obtain the creep curve under different loads. The creep curves under each loading was obtained by the coordinate translation method, as shown in Fig. 6. When the loading time *t* = 400 min, the creep strain *ε*_*c*_ = 0. The creep curves of the clayey soil specimens have obvious nonlinear characteristics, creep deformation increases gradually with the increase of time, but the increase rate generally shows a decreasing trend. It can also be seen from Fig. 6 that the amount of creep increases gradually as the load increases.

### Analysis of isochronous stress–strain curves

**Fig. 7:**
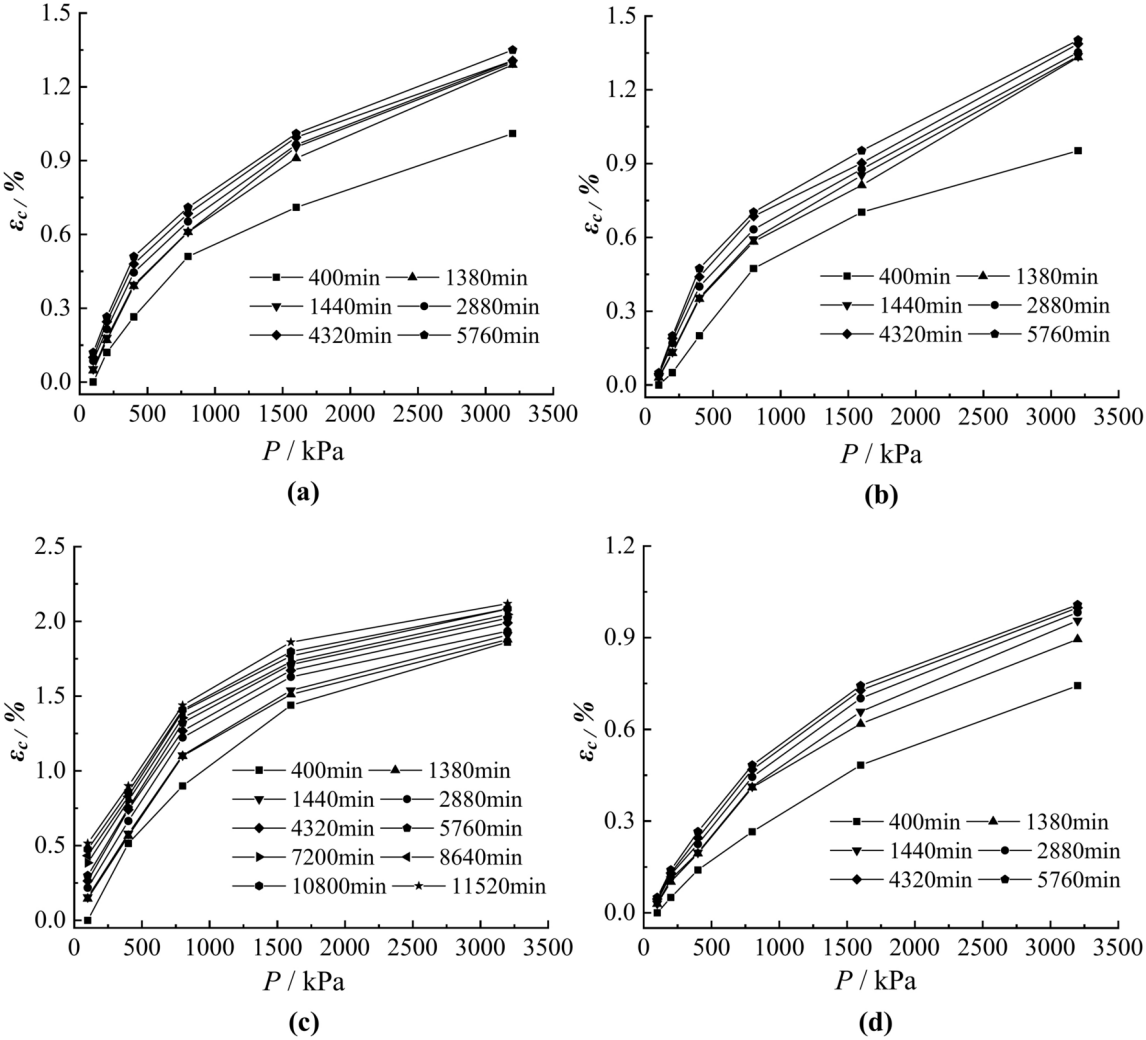
Isochronous stress–strain curves of the soil samples N1~N4. (**a**) N1 (**b**) N2 (**c**) N3 (**d**) N4.

The isochronous stress–strain curves of soil samples N1~N4 were obtained by using the Boltzmann superposition principle, as shown in Fig. 7, the curves under different loading have similar shapes, and the strain increases with the increase of time for the same load, but the isochronous curves gradually get close to form a curve cluster. The creep deformation increases with the increase of stress, and the stress–strain shows a nonlinear relationship, which should be taken into account when constructing the creep models.

## Creep model

### Classical rheological models

The basic elements commonly used to describe the rheology of materials include Hooke elastomer, Newton viscous body and St. Venant plastic body. By combining these basic elements, the various creep models can be obtained, which can be used to present the relationship between stress, strain and time of rock and soil mass. The classical models and mathematical expressions are shown in Table 2. Because the basic components of these models have clear physical meanings, which is convenient for the in-depth analysis of the research object, this type of models are more widely used in the creep study of rock and soil mass.

**Table 2:** Classical creep models and calculation formulas.

Number	Model name	Creep equation	Model structure
1	Kelvin model	$\varepsilon = \frac{\sigma }{EH}\left( {1 - e^{{ - \left( {\frac{{E_{H} }}{\eta }} \right)t}} } \right)$	
2	Maxwell model	$\varepsilon = \sigma \left( {{t \mathord{\left/ {\vphantom {t \eta }} \right. \kern-0pt} \eta } + {1 \mathord{\left/ {\vphantom {1 {E_{H} }}} \right. \kern-0pt} {E_{H} }}} \right)$	
3	Merchant model	$\varepsilon = \frac{\sigma }{EH} + \frac{\sigma }{EK}\left( {1 - e^{{ - \left( {\frac{EK}{\eta }} \right)t}} } \right)$	
4	Burgers model	$\varepsilon = \sigma \left[ {\frac{1}{EH} + \frac{t}{{\eta_{1} }} + \frac{1}{EK}\left( {1 - e^{{ - \left( {\frac{EK}{\eta }} \right)t}} } \right)} \right]$	

*E*_*H*_ is Hooke’s elastic modulus, MPa; *E*_*K*_ is Kelvin body’s elastic modulus, MPa; *η* is Kelvin body’s viscosity coefficient, MPa⋅min; *η*_1_ is Maxwell body’s viscosity coefficient, MPa⋅min.

### An improved nonlinear creep model

The classical creep models above have the following shortcomings: (1) They are not effectively combined with the consolidation theory of soil mechanics, but based on the viscosity, elasticity and plasticity of general materials, which cannot reflect the deformation caused by consolidation and drainage of clayey soil, and the results calculated by the above models are inconsistent with the actual observation results; (2) They cannot reflect the three stages of instantaneous deformation, primary consolidation and secondary consolidation (creep) in the consolidation theory; (3) When t = 0, the creep strain is theoretically 0, however, the results calculated by the above models all have transient strains, such as the Burgers model, which calculates the creep strain at t = 0 to be $\varepsilon = {\sigma \mathord{\left/ {\vphantom {\sigma {E_{H} }}} \right. \kern-0pt} {E_{H} }}$, which is inconsistent with reality. Considering the creep behavior of clayey soil in land subsidence areas, the NCE_CS nonlinear creep model is proposed by replacing elastic elements in Merchant model with power function elements and improving Kelvin body in Merchant model to consider the beginning time of creep in consolidation theory.

#### Improvement of Kelvin model

Kelvin model consists of a Hooke elastomer and a Newton viscous body in parallel (Table 2). When time *t* → ∞, the strain tends to be a constant, reflecting the property of steady-state creep of the soil. The strain after unloading takes some time to decrease to 0, reflecting the nature of elastic aftereffects. The Kelvin model can be expressed as^26^

4 $$\sigma_{K} = E_{H} \varepsilon_{kc} + \eta \dot{\varepsilon }_{kc}$$

where ${\varepsilon }_{kc}$ is the creep of clayey soil under load $\sigma_{K}$, *E*_*H*_ is Hooke elastic modulus, *η* is the viscosity coefficient in MPa·min, and $\dot{\varepsilon }_{kc}$ is the creep that occurs per unit time and reflects the speed of creep deformation.

In the strain–time natural logarithm coordinate system, the creep rate can be expressed as:

5 $$\dot{\varepsilon }_{kc}  = \frac{{d\varepsilon_{kc} }}{dt} = \frac{{\varepsilon_{kc} \left( t \right) - \varepsilon_{kc} \left( {t_0} \right)}}{{\left( {\ln \left( t \right) - \ln \left( {t_0} \right)} \right) \cdot \min }}$$

where *t* is any time in the creep phase; *t*_0_ is the start time of creep stage, here 400 min is taken; min is the time unit minute.

Substituting Eq. 5 into Eq. 4:

6 $$\sigma_{K} = E_{H} \varepsilon_{kc} + \eta \frac{{d\varepsilon_{kc} }}{{d\ln \left( {{t \mathord{\left/ {\vphantom {t {t_{0} }}} \right. \kern-0pt} {t_{0} }}} \right) \cdot \min }}$$

Equation 7 is derived by transposition of Eq. 6:

7 $$\frac{{d\varepsilon_{kc} }}{{\sigma_{K} - E_{H} \varepsilon_{kc} }} = \frac{{d\ln \left( {{t \mathord{\left/ {\vphantom {t {t_{0} }}} \right. \kern-0pt} {t_{0} }}} \right) \cdot \min }}{\eta }$$

When *t* = *t*_0_, creep begins, and let $\varepsilon_{kc} \left( {t_{0} } \right) = 0$; By integrating both sides of Eq. 6 in the range of *t*_0_ to *t*, Eq. 8 can be obtained:

8 $$- \frac{1}{{E_{H} }}\ln \left( {\frac{{\sigma_{K} - E_{H} \varepsilon_{kc} \left( t \right)}}{{\sigma_{K} }}} \right) = \frac{1}{\eta }\ln \left( {\frac{t}{{t_{0} }}} \right)\min$$

The simplification of Eq. 8 is as follows:

9 $$\varepsilon_{kc} \left( t \right) = \frac{{\sigma_{K} }}{{E_{H} }}\left( {1 - e{}^{{ - \frac{{E_{H} }}{\eta }\ln \left( {{t \mathord{\left/ {\vphantom {t {t_{0} }}} \right. \kern-0pt} {t_{0} }}} \right) \cdot \min }}} \right)$$

By sorting out Eq. 9, the improved Kelvin equation is obtained:

10 $$\varepsilon_{kc} \left( t \right) = \frac{{\sigma_{K} }}{{E_{H} }}\left( {1 - \left( {{\raise0.7ex\hbox{$t$} \!\mathord{\left/ {\vphantom {t {t_{0} }}}\right.\kern-0pt} \!\lower0.7ex\hbox{${t_{0} }$}}} \right)^{{ - \frac{{E_{H} }}{\eta } \cdot \min }} } \right)$$

The improved Kelvin creep model is more concise and is able to take into account the time of occurrence of the secondary consolidation (creep) phase in consolidation theory, making the division between creep and primary consolidation deformation clearer.

#### Power function element

**Fig. 8:**
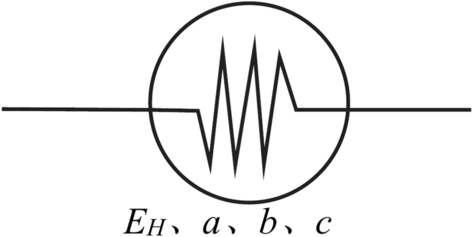
Power function element.

The creep of clayey soil can be highly fitted by a power function^15^. The proposed power function element, as shown in Fig. 8, is similar to a viscoelastic element and can reflect the nonlinear behavior of creep. Its equation can be expressed as Eq. 11.

11 $$\sigma = \frac{{E_{H} }}{{a\left( {{t \mathord{\left/ {\vphantom {t {t_{0} + b}}} \right. \kern-0pt} {t_{0} + b}}} \right)^{c} }}\varepsilon_{pc}$$

Equation 11 is expressed in stress form as Eq. 12:

12 $$\varepsilon_{pc} = \frac{\sigma }{{E_{H} }}a\left( {{t \mathord{\left/ {\vphantom {t {t_{0} + b}}} \right. \kern-0pt} {t_{0} + b}}} \right)^{c}$$

where $\varepsilon_{pc}$ is the calculated creep, $\sigma$ is the magnitude of the load, and *a*, *b*, *c* are model parameters.

Using this power function element, the creep at the moment *t* = *t*_0_ can be calculated to be 0, and the effects of variable dimension are eliminated by using *t*/*t*_0_ as the creep time.

#### NCE_CS model

**Fig. 9:**
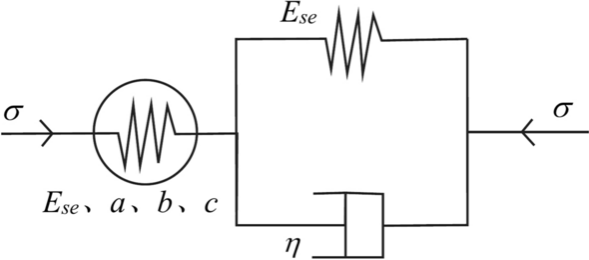
NCE_CS nonlinear creep model.

Combined with the one-dimensional creep test results of clayey soil in the land subsidence area of Cangzhou, the power function element replaces the Hooker’s elastic element in the Merchant model and is connected in series with the improved Kelvin body to obtain the nonlinear creep NCE_CS model, which is schematically shown in the model structure in Fig. 9.

The NCE_CS model is expressed as Eq. 13:

13 $$\varepsilon_{c} \left( t \right) = \frac{\sigma }{{E_{se} }}a\left( {{t \mathord{\left/ {\vphantom {t {t_{0} + b}}} \right. \kern-0pt} {t_{0} + b}}} \right)^{c} + \frac{\sigma }{{E_{se} }}\left[ {1 - \left( {{\raise0.7ex\hbox{$t$} \!\mathord{\left/ {\vphantom {t {t_{0} }}}\right.\kern-0pt} \!\lower0.7ex\hbox{${t_{0} }$}}} \right)^{{ - \frac{{E_{se} }}{\eta } \cdot \min }} } \right]$$

Considering that the isochronous stress–strain curves (Fig. 7) have nonlinear characteristics, the modulus $E_{H}$ in Eqs. 10 and 12 is different from the elastic modulus $E_{se}$ in Eq. 13, which varies with the change of loading and can be calculated from the segmented compression modulus on the *e-*log(*P*) curves.

14 $$E_{se} = \left( {1 - \frac{{2\mu^{2} }}{1 - \mu }} \right)E_{si}$$

15 $$E_{si} = \frac{{\sigma_{i + 1} - \sigma_{i} }}{{e_{i} - e_{i + 1} }}\left( {1 + e_{0} } \right)$$

where $\varepsilon_{c} \left( t \right)$ is the creep under the stress $\sigma$; * a*, *b*, *c* are model parameters;$E_{se}$ is the segmental modulus at each level of loading; $E_{si}$ is the segmental compression modulus under load level *i*; $\sigma_{i + 1}$ and $\sigma_{i}$ are the magnitude of the load of the (*i* + 1)th and *i*-th level, respectively; $e_{i + 1}$ and $e_{i}$ are the pore ratio under the load of the (*i* + 1)th and *i*-th level, respectively; $e_{0}$ is the initial pore ratio; *μ* is the Poisson’s ratio.

The mechanical behavior of clayey soils exhibits strain rate sensitivity, which means that their strength and deformation vary with different loading rates. Elastoplastic constitutive models usually introduce strain rate effects indirectly through over-stress theory, thus limiting their applicability. Unlike elastoplastic models^41,42^, the proposed model not only captures the time-dependent deformation under long-term loads, but also directly reflects the strain rate sensitivity through the viscous Kelvin element.

### Determination of model parameters

The parameter solution of NCE_CS model is actually a multivariate function parameterization problem, and the genetic algorithm can find the global solution more accurately, which is very suitable for the optimization of multivariate function parameterization. The NCE_CS model includes four parameters (*a*, *b*, *c*, *η*), which can be solved by the genetic algorithm through the module sko.GA, which is written in Python language. The algorithm is a simulation of the biological evolution process, taking creep $\varepsilon_{c} \left( t \right)$ as the objective function (Eq. 13), time *t* and stress *σ* as variables, and optimizing the parameters by random search.

**Fig. 10:**
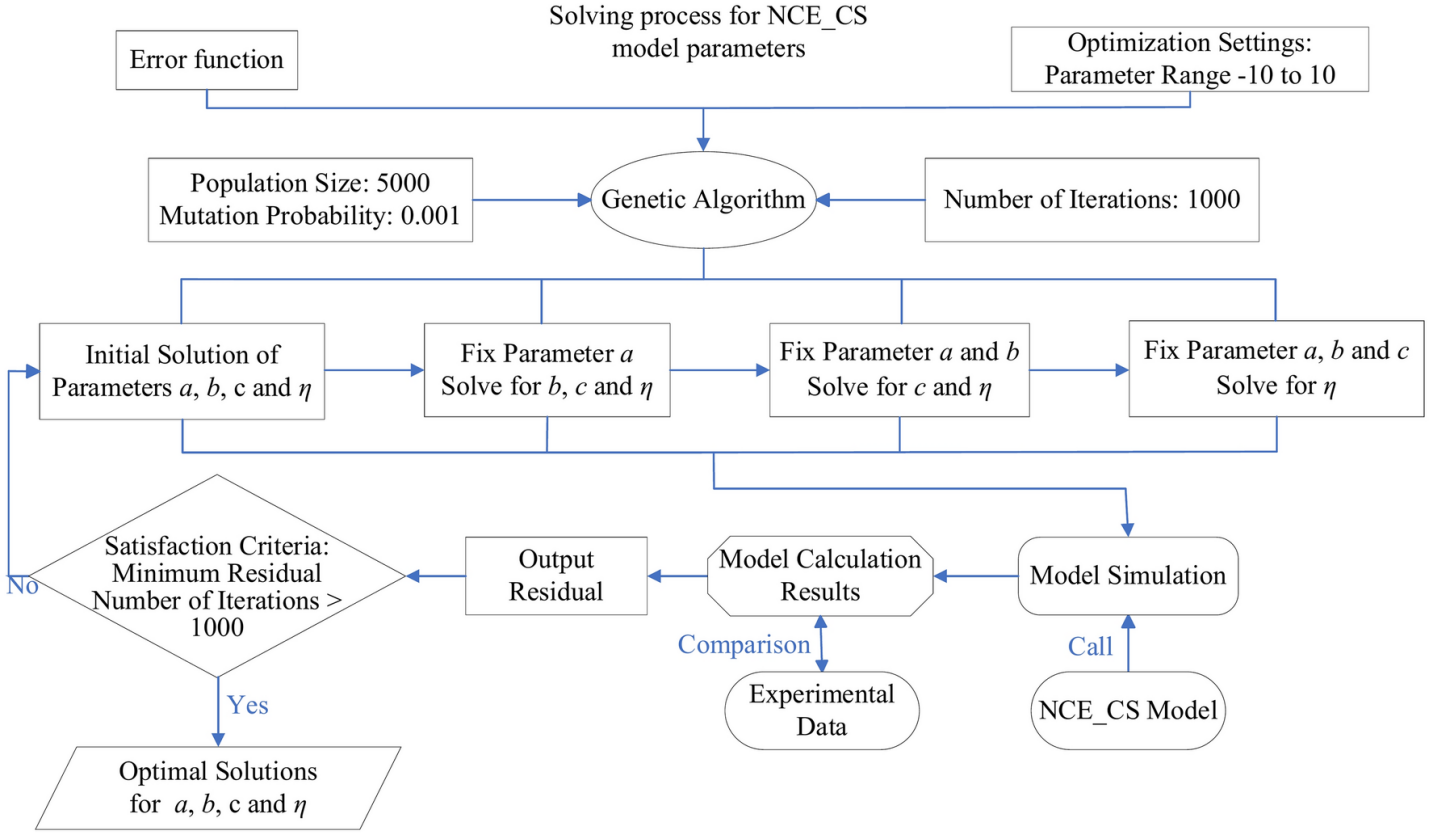
Flow chart of genetic algorithm solution.

The solution steps are as follows (Fig. 10): (1) Initialize the population; the population number is set to 5000 in this paper. (2) The individuals are assessed to determine their fitness. (3) Selection and crossover; the individuals are selected according to the fitness, then the selected individuals are crossed and new individuals are produced. (4) Mutation; the diversity of samples is increased through variation, and the mutation probability is set at 0.001 in this paper. (5) Repeat the above steps; the calculation is stopped when the maximum number of iterations (1000 times in this paper) is reached or the optimal solution is obtained. The modulus *E*_*se*_ is obtained by the consolidation test data and Eqs. 14 and 15. The calculation results of the model parameters are shown in Table 3.

**Table 3:** Calculation results of the parameters of NCE_CS model for N1–N4.

Soil sample number	*P* (MPa)	*W*	Model parameters				
			*a*	*b*	*c*	$\eta$ (MPa min)	*E*_*se*_ (MPa)
N1	0.1	8.677	0.285	0.401	0.733	2.955	2.665
	0.2	4.339	2.078	0.423	0.332	2.976	4.577
	0.4	2.169	4.159	1.543	0.222	2.807	6.979
	0.8	1.085	6.774	1.781	0.181	2.544	12.419
	1.6	0.542	11.84	6.466	0.074	2.358	27.352
	3.2	0.271	9.523	7.82	0.128	2.596	35.985
N2	0.1	11.860	0.074	0.371	0.789	2.975	3.026
	0.2	5.930	1.233	0.654	0.451	2.672	4.946
	0.4	2.965	3.828	1.305	0.238	2.67	8.831
	0.8	1.482	6.691	2.52	0.249	2.212	15.435
	1.6	0.741	8.75	3.98	0.105	2.655	21.798
	3.2	0.371	14.186	7.902	0.085	2.845	46.096
N3	0.1	12.407	2.053	0.378	0.677	2.655	4.276
	0.4	3.102	4.606	0.604	0.282	2.183	5.346
	0.8	1.551	6.480	2.279	0.183	2.120	6.481
	1.6	0.775	10.573	4.299	0.114	2.144	11.946
	3.2	0.388	15.461	6.558	0.076	2.577	26.289
N4	0.1	14.609	0.042	0.316	0.773	2.979	2.753
	0.2	7.305	2.511	0.616	0.463	2.648	11.768
	0.4	3.652	3.643	1.167	0.282	2.654	13.364
	0.8	1.826	5.350	2.013	0.175	2.629	15.859
	1.6	0.913	7.714	3.561	0.106	2.674	24.757
	3.2	0.457	11.387	5.594	0.069	2.461	47.252

*W* is the load action ratio, see Section “*Dependence of parameters a, b and c on consolidation pressure*” for definition.

**Fig. 11:**
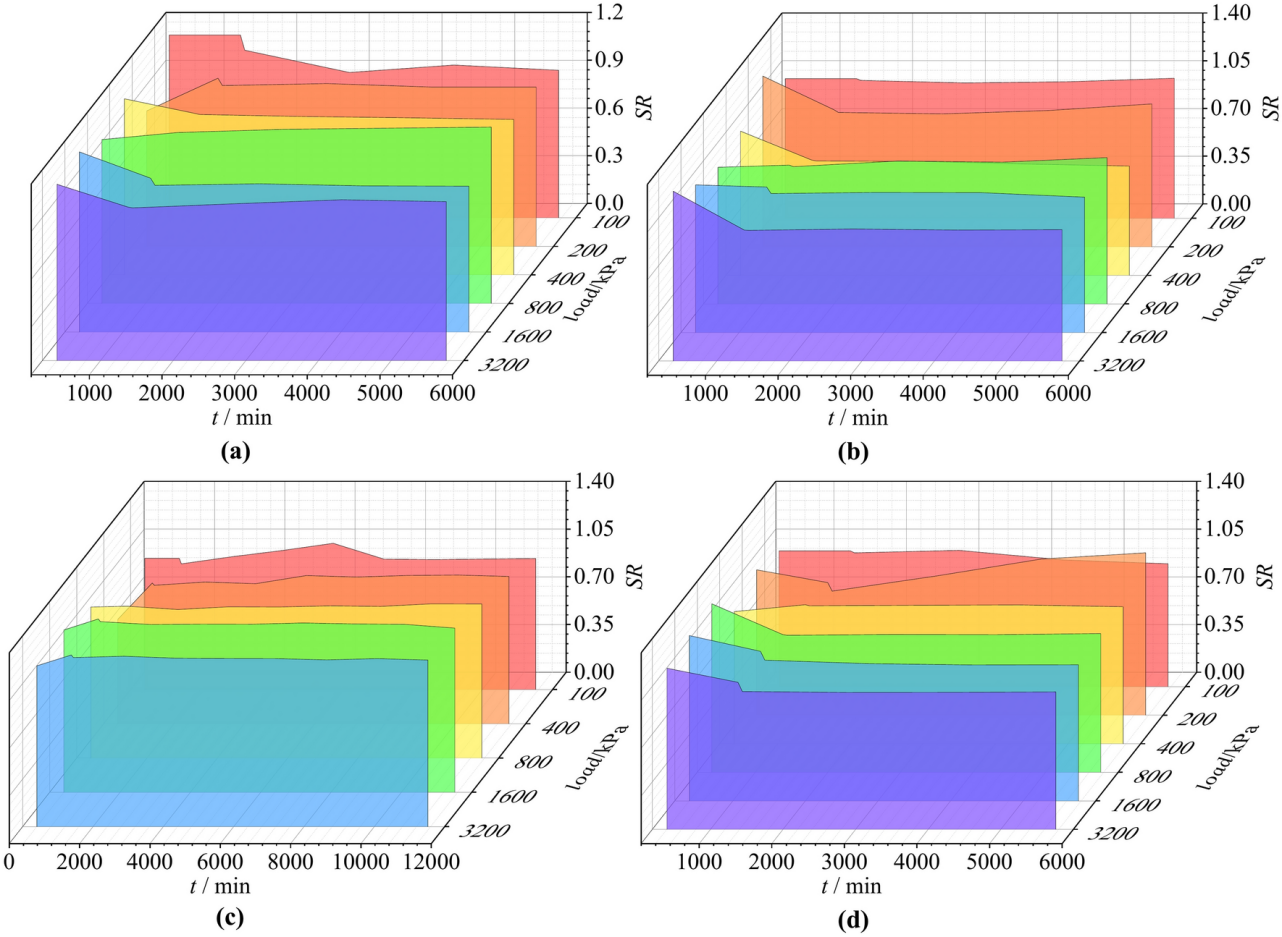
Changes of *SR* values with time under different loads. (**a**) N1 (**b**) N2 (**c**) N3 (**d**) N4.

The parameters in Table 3 are substituted into Eq. 13, and the creep calculation results and *SR* under various loads are obtained. $SR = \frac{{\varepsilon_{c - cal} \left( t \right)}}{{\varepsilon_{c - tes} \left( t \right)}}$ is a ratio between the calculated value $\varepsilon_{c - cal} \left( t \right)$ and the testing value $\varepsilon_{c - tes} \left( t \right)$ at any time point, and the ratio can reflect the accuracy of the calculated results. The variation of *SR* values of specimens N1~N4 with time under different loads is shown in Fig. 11, from which it can be seen that the *SR* values are generally large at the initial moment and when the load is small, and the remaining *SR* values varying around 1.0. The maximum value of *SR* is 1.27 and the minimum value is 0.77, 85.1% of the *SR* values are between 0.8 and 1.2, which indicates that the model predicts well.

#### Dependence of parameters *a*, *b* and *c* on consolidation pressure

**Fig. 12:**
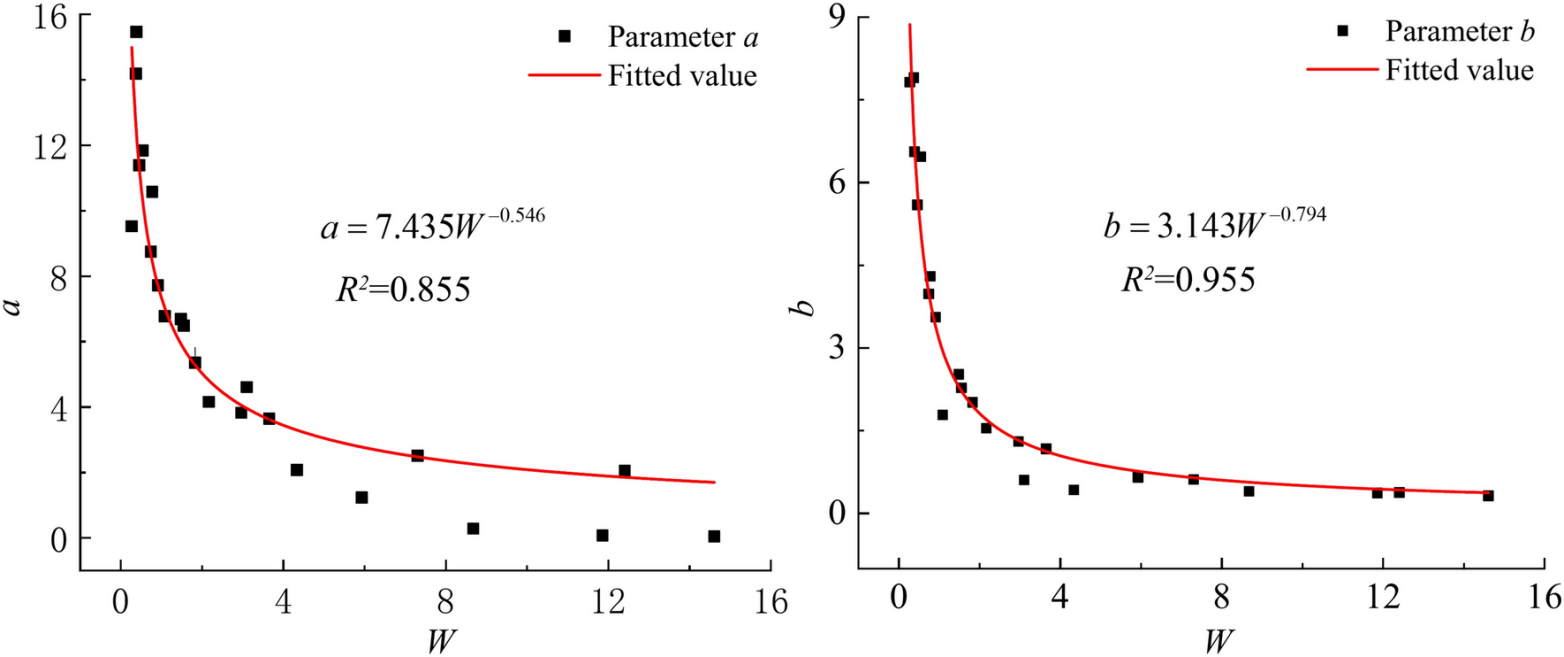
Relations between parameters *a*, *b* and *W.*

In order to make the model more widely applicable, this paper tries to find the relationship between the model parameters and other physico-mechanical indexes. Imitating the definition of the overconsolidation ratio, the load action ratio *W* is defined as the ratio of the soil’s self-weight stress to the total pressure currently borne by the soil. The relationship between parameters *a* and *b* and the load action ratio *W* is shown in Fig. 12, approximately in the form of power function, indicating that the parameters *a* and *b* decrease with the increase of *W*. When *W* is between 0 and 4.0, the rate of decrease of parameters *a* and *b* is large, and the rate after *W* > 4 is small. The nonlinear curve fitting function in Origin software was utilized to fit the experimental data, and the relationship between parameters *a*, *b* and *W* is shown in Eqs. 16 and 17, whose coefficients of determination are 0.855 and 0.955, respectively, indicating a good fitting effect.

16 $$a = 7.435W^{ - 0.546}$$

17 $$b = 3.143W^{ - 0.794}$$

**Fig. 13:**
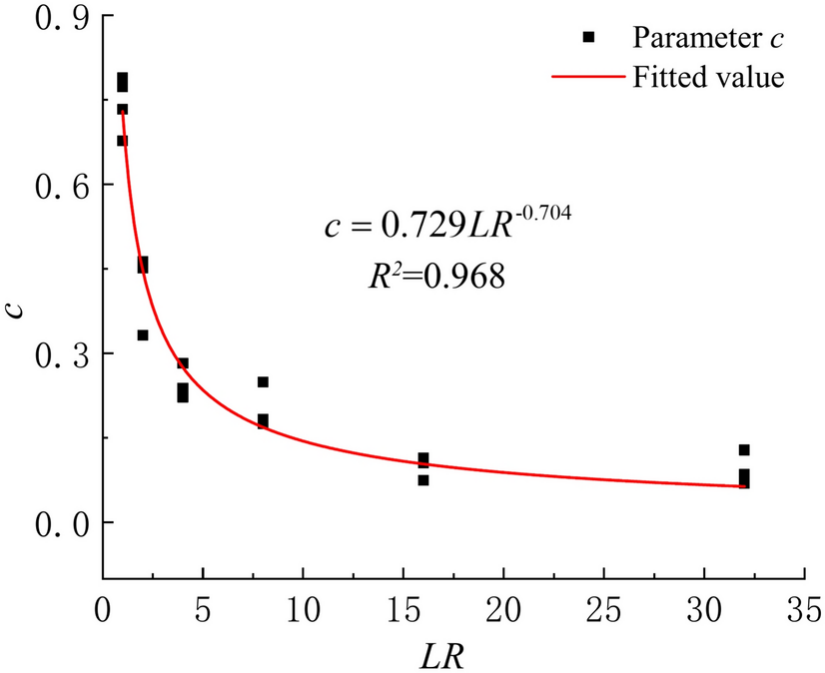
Relations between parameters *c* and *LR.*

To make the variables dimensionless, the load $$\sigma$$ divided by standard atmospheric pressure $${\text{P}}_{{\text{a}}}$$ ($$LR = {\raise0.7ex\hbox{$\sigma $} \!\mathord{\left/ {\vphantom {\sigma {{\text{P}}_{{\text{a}}} }}}\right.\kern-0pt} \!\lower0.7ex\hbox{${{\text{P}}_{{\text{a}}} }$}}$$) is defined as the load-to-pressure ratio. The parameter *c* decreases with the increase of *LR*, which is approximately in the form of a power function (Fig. 13). The relationship between parameter *c* and *LR* can be expressed in Eq. 18 with a coefficient of determination of 0.968.

18 $$c = 0.729LR^{{{ - 0}{\text{.704}}}}$$

#### The influence of parameter $\eta$ on the calculation results

The analysis shows that the regularity of the relationship between model parameters such as viscosity and load-action ratio and physical property indices is poor. It is necessary to analyze the effect of $\eta$ on calculation results of the creep. The values of $\eta$ in Table 3 range from 2.120 to 2.979, with a mean value of 2.6. The value of $\eta$ can be taken as 2.0, 2.2, 2.4, 2.8, 3.0 and 3.2, respectively, and the creep is calculated according to Eq. 13, and then compared with the creep calculated at $\eta$ = 2.6. The error is calculated according to Eq. 19.

19 $$P_{er} = \frac{1}{N}\sum\limits_{i = 1}^{N} {\frac{{\left| {\varepsilon_{\eta ,i} - \varepsilon_{2.6,i} } \right|}}{{\varepsilon_{2.6,i} }}} \times 100\%$$

where $P_{er}$ is the average of the creep calculation errors for different values of $\eta$. *N* is total number of comparisons;$\varepsilon_{\eta ,i}$ is the creep calculated by different $\eta$ at the *i-*th comparison; $\varepsilon_{2.6,i}$ is the creep calculated with $\eta = 2.6$ at the *i-*th comparison.

**Fig. 14:**
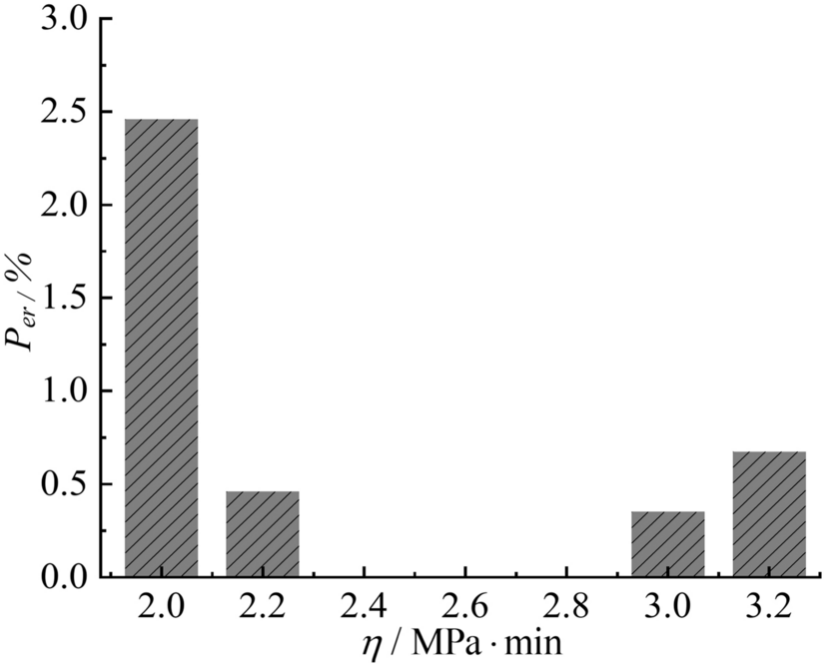
Errors in creep calculation (Eq. 19) when $\eta$ takes different values.

As the deviation from $\eta = 2.6$ increases, the error $P_{er}$ of creep calculation also increases, up to a maximum of only 2.46% (Fig. 14). When $\eta = 2.4$ and $\eta = 2.8$, the error is close to 0. This indicates that the variation of $\eta$ in the range of 2.0~3.2 has little effect on the creep calculation results, and so let $\eta = 2.6$.

Equations 16, 17, 18 and $\eta = 2.6$ are substituted into Eq. 13 to obtain the expression of the nonlinear creep NCE_CS model (Eq. 20):

20 $$\varepsilon_{c} \left( t \right) = \frac{\sigma }{{E_{se} }}\left( {7.435W^{ - 0.546} } \right)\left[ {{t \mathord{\left/ {\vphantom {t {t_{0} }}} \right. \kern-0pt} {t_{0} }} + \left( {3.143W^{ - 0.794} } \right)} \right]^{{0.729LR^{{ - {0}{\text{.704}}}} }} + \frac{\sigma }{{E_{se} }}\left[ {1 - \left( {{\raise0.7ex\hbox{$t$} \!\mathord{\left/ {\vphantom {t {t_{0} }}}\right.\kern-0pt} \!\lower0.7ex\hbox{${t_{0} }$}}} \right)^{{ - \frac{{E_{se} }}{\eta } \cdot \min }} } \right]$$

### Verification and application of the model

#### Comparison of predicted results with testing data

**Fig. 15:**
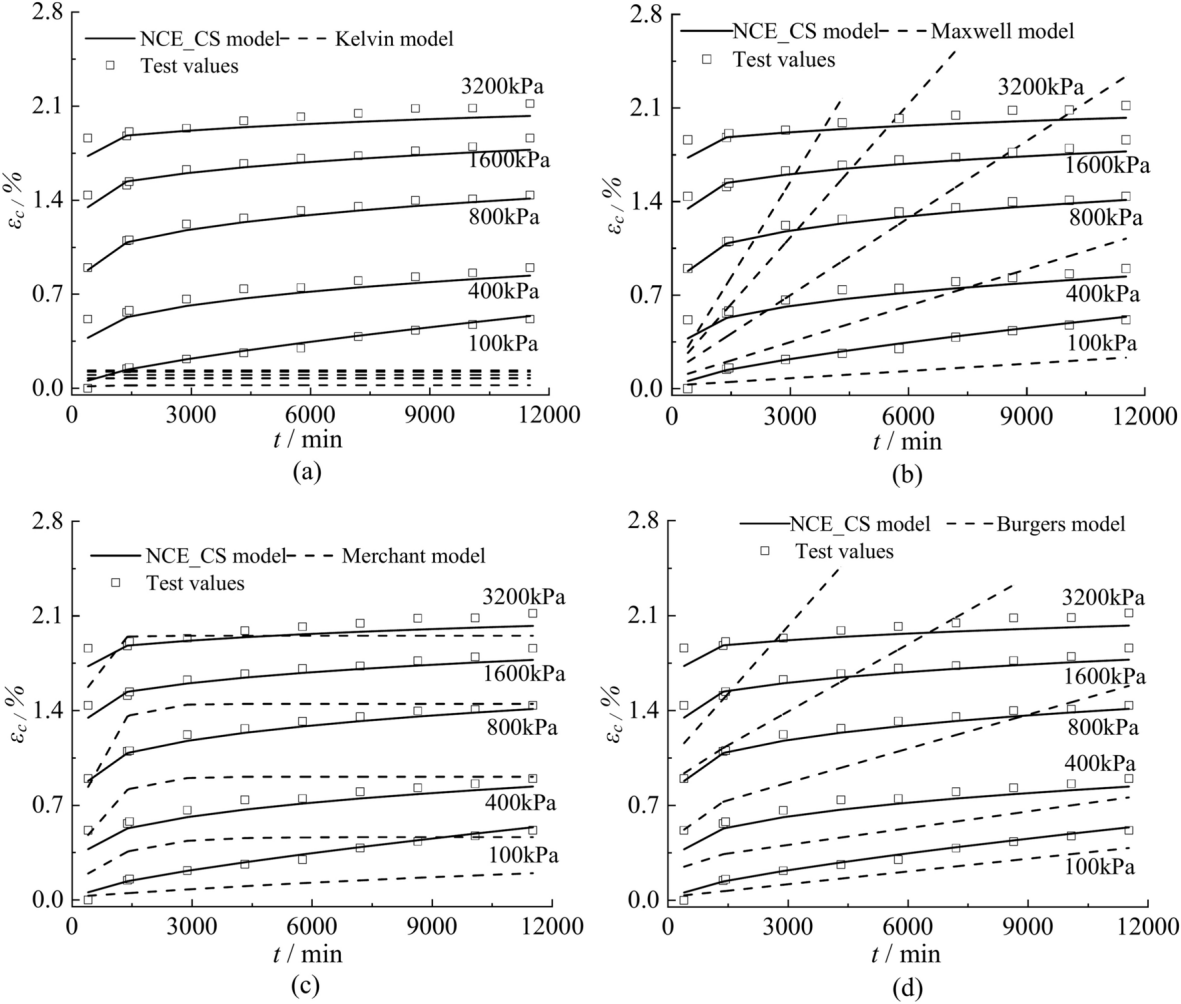
Creep curves calculated by different creep models versus test data.

**Fig. 16:**
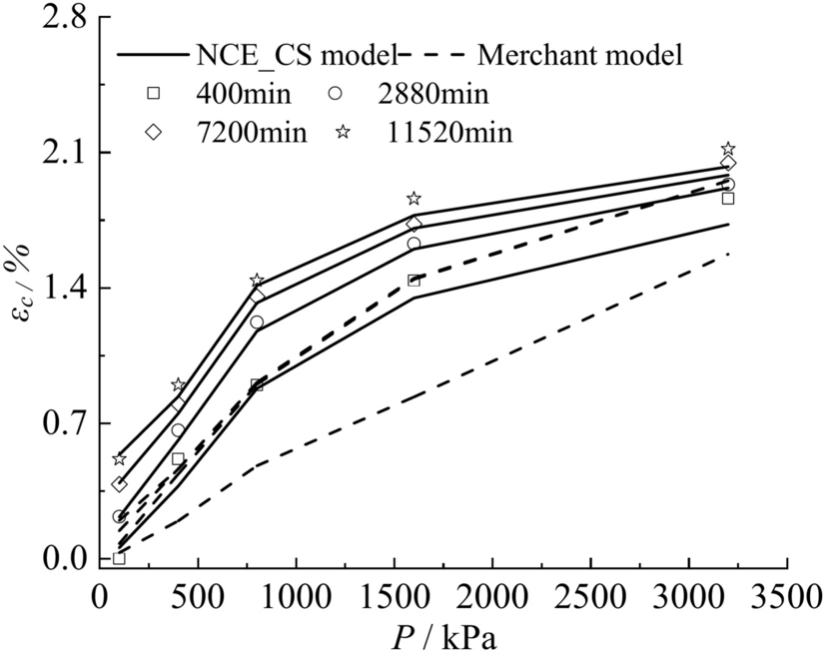
Stress–strain curves calculated by NCE_CS model, Merchant model versus test data.

In order to verify the reliability of the NCE_CS model, the calculated results of the NCE_CS model were compared with the test data and the calculated results of the Kelvin model, Maxwell model, Merchant model, and Burgers model, respectively, in terms of the creep curve and the isochronous stress–strain curve. The comparison results of specimen N3 are shown in Figs. 15 and 16. Based on the experimental data, genetic algorithm was used to find the parameters of the above four classical creep models, as shown in Table 4.

**Table 4:** Parameters of the classical creep models for the soil sample N3.

Soil sample number	Model name	*P* (MPa)	*E*_*H*_ (MPa)	*E*_*K*_ (MPa)	$\eta$ (MPa min)	$\eta_{1}$ (MPa min)
N3	Kelvin model	0.1	4.276		1673.397	
		0.4	5.346		65.737	
		0.8	6.481		66.838	
		1.6	11.946		61.922	
		3.2	26.289		185.513	
	Maxwell model	0.1	4.276		5517.496	
		0.4	5.346		4398.110	
		0.8	6.481		4163.015	
		1.6	11.946		4810.323	
		3.2	26.289		6731.664	
	Merchant model	0.1	4.276	0.274	4852.449	
		0.4	5.346	1.026	1104.430	
		0.8	6.481	1.015	671.624	
		1.6	11.946	1.216	637.191	
		3.2	26.289	1.746	444.128	
	Burgers model	0.1	4.276	0.005	7465.929	5518.263
		0.4	5.346	1.904	558.007	9721.543
		0.8	6.481	1.626	482.986	9543.877
		1.6	11.946	2.102	242.771	9654.988
		3.2	26.289	3.539	7.202	9632.110

Figure 15 shows that the creep curve of the NCE_CS model is more consistent with the test data, and the creep shows a nonlinear steady state development trend. The calculation results of the Merchant model are obviously smaller than the test data, and the creep growth is faster at the beginning, and then increases slowly at the later stage of the creep (Fig. 15c). The calculation results of the Kelvin model are very different from the test data, and the results evolve into a nearly horizontal straight line with the increase of time (Fig. 15a). The calculation results of Maxwell model and Burgers model have a big difference with the test data, and the calculation results show a linear increase with the increase of time, and the slope increases with the increase of load (Fig. 15b, d). Since the creep curves calculated by the Kelvin model, Maxwell model, and Burgers model differ significantly from the experimental data, only the isochronous stress–strain curves of the NCE_CS model and the Merchant model are compared with the experimental data. As shown in Fig. 16, there is a discrepancy between the results calculated by the Merchant model and the experimental data, and the shapes of the curves are also inconsistent. The creep isochronous stress–strain curves calculated by NCE_CS model are in good agreement with the experimental data, which indicates that the NCE_CS model is able to describe the creep characteristics of clayey soil in Cangzhou.

#### Application of NCE_CS model

**Fig. 17:**
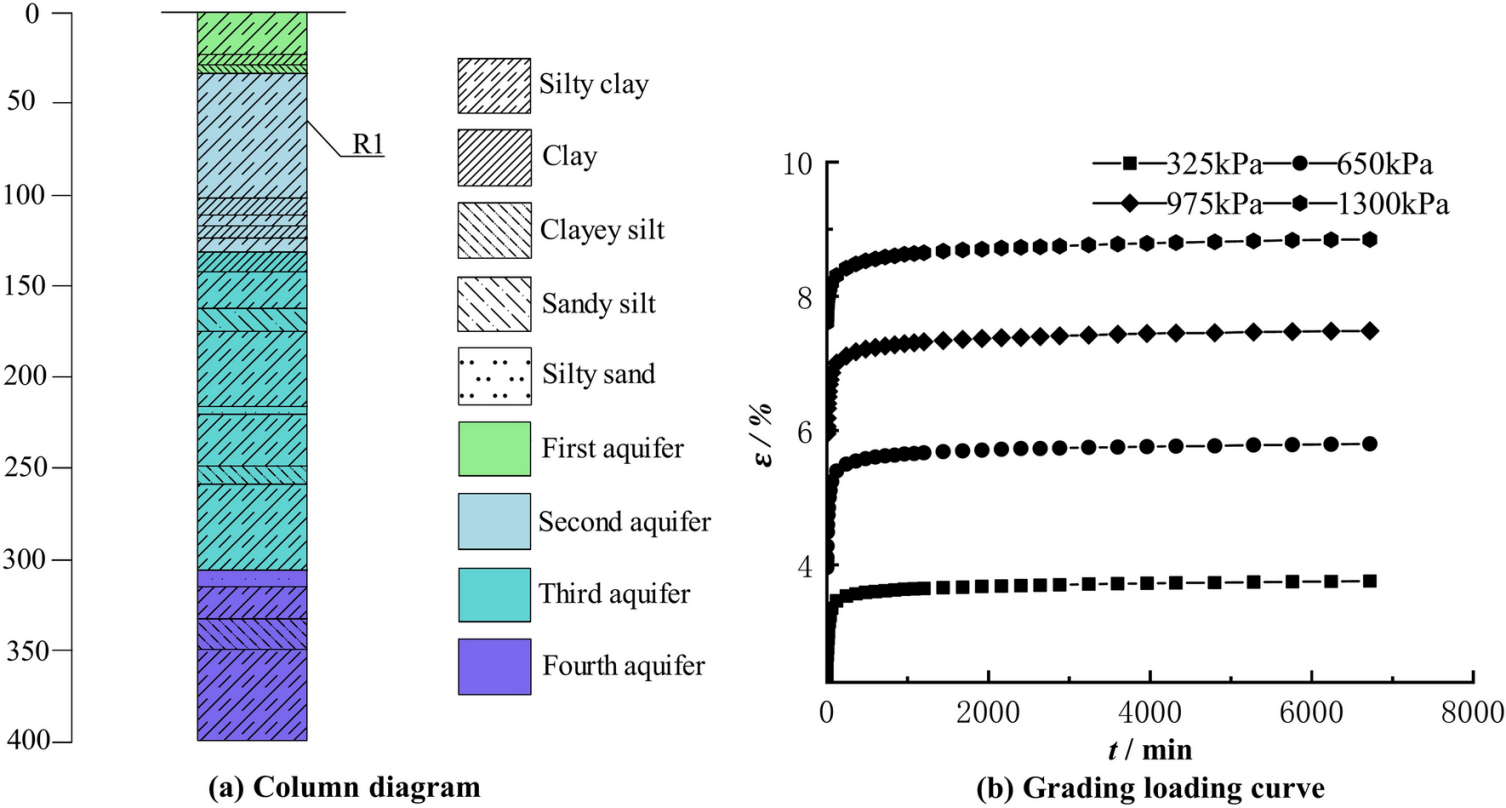
Borehole histogram and stepwise loading creep curves in Renqiu.

In order to investigate the causes of land subsidence, hydrogeological drilling and sampling were conducted in Renqiu City in northwest of Cangzhou (Fig. 2b). The drilling reached a depth of 400.0 m (Fig. 17a), in which clayey soil samples were collected. The basic geotechnical test and compression creep test were carried out on the clayey soil sample R1 buried at a depth of 58.86 m. The physical indexes of the soil sample R1 are shown in Table 5. The compression creep tests were carried out in a MIS-232 high pressure consolidation instrument from Japan with applied loads of 0.325 MPa, 0.65 MPa, 0.975 MPa, and 1.3 MPa, and the loading duration was 112 h. The stepwise loading strain–time curves (Fig. 17b) of specimen R1 are similar to those of specimens N1~N4, and the creep deformation under different loads shows a steady state development trend as time increases.

**Table 5:** Basic physical properties of the undisturbed soil sample R1 in Renqiu.

Soil sample number	Depth (m)	Water content *w* (%)	Specific gravity *G*_s_	Void ratio *e*	Liquid limit *W*_L_ (%)	Plastic limit *W*_P_ (%)	Plasticity index *I*_P_	Saturation *S*_r_ (%)	Gravity stress (kPa)
R1	58.86	25.9	2.73	0.727	35.8	20.2	15.6	97.2	780.0

By calculating and analyzing the test data, the values of *E*_se_, *W* and *LR* at all levels of loading were obtained (Table 6). The value of $\eta$ is still taken as 2.6.

**Table 6:** Values of variables at each level of loading in the NCE_CS model for R1.

Soil sample number	Loads (MPa)	*E*_se_ (MPa)	*W*	*LR*
R1	0.325	12.22	2.4	3.25
	0.65	13.871	1.2	6.5
	0.975	15.373	0.8	9.75
	1.3	16.94	0.6	13.0

Comparing the clayey soils of Renqiu City and Cangzhou City, due to differences in composition, deposition environment, and groundwater level, the power function relationship between parameters *a*, *b*, *c* and *W*, *LR* in Eq. 20 remains unchanged, but the values of the constant terms in Eq. 16, 17, and 18 will be changed. The parameters of the NCE_CS model were solved using genetic algorithm, and Eq. 21 was finally obtained.

21 $$\varepsilon_{c} \left( t \right) = \frac{\sigma }{{E_{se} }}\left( {5.92W^{ - 0.947} } \right)\left[ {{t \mathord{\left/ {\vphantom {t {t_{0} }}} \right. \kern-0pt} {t_{0} }} + \left( {0.1W^{ - 0.1} } \right)} \right]^{{0.799LR^{{ - {0}{\text{.893}}}} }} + \frac{\sigma }{{E_{se} }}\left[ {1 - \left( {{\raise0.7ex\hbox{$t$} \!\mathord{\left/ {\vphantom {t {t_{0} }}}\right.\kern-0pt} \!\lower0.7ex\hbox{${t_{0} }$}}} \right)^{{ - \frac{{E_{se} }}{\eta } \cdot \min }} } \right]$$

**Fig. 18:**
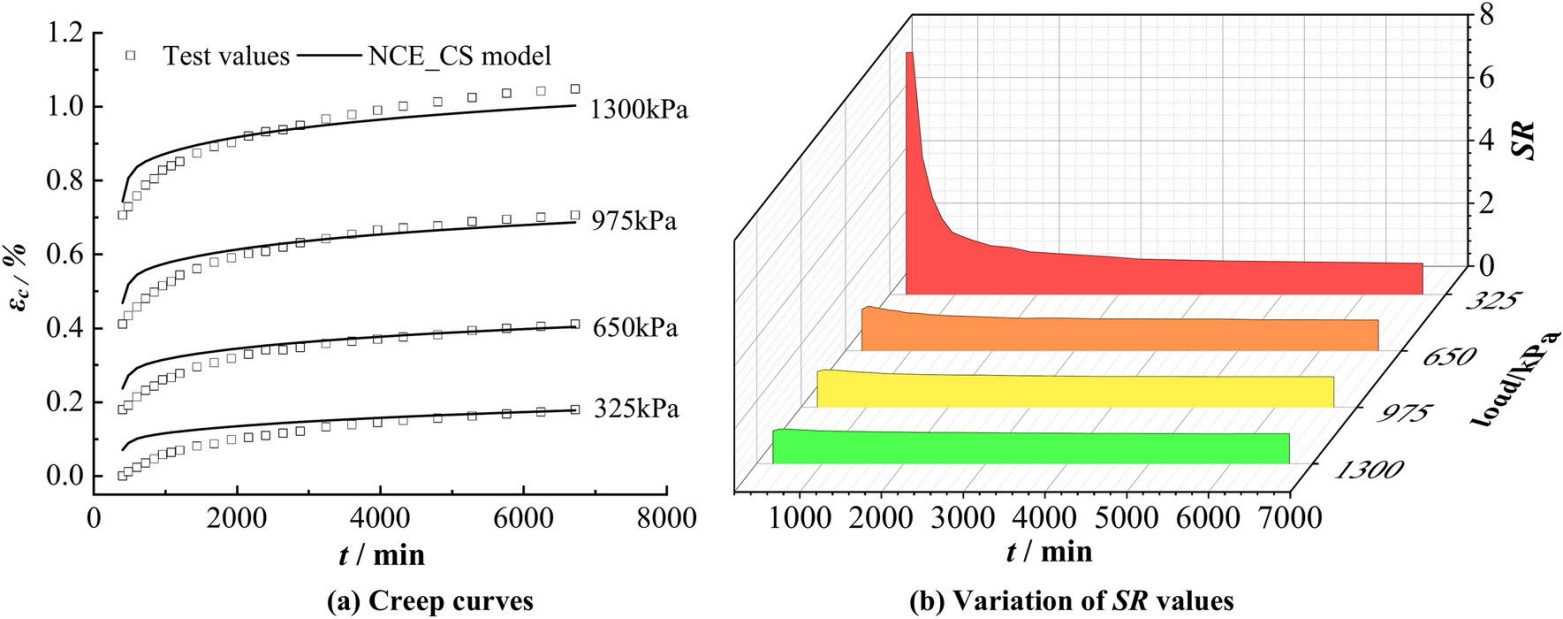
Calculation results of NCE_CS model versus test data of clayey soil in Renqiu.

The creep curves (Fig. 18a) at all levels of loading were calculated using Eq. 21, which are overall consistent with the test data, with a large deviation only in the initial stage at a load of 0.325 MPa. Figure 18b shows that the maximum value of *SR*, which reflects the calculation accuracy, is at the initial stage of creep under the load of 0.325 MPa. The maximum value of *SR* is 7.694, the minimum value is 0.957, and the percentage of *SR* values between 0.9 and 1.2 is 78.95%, which indicates that the NCE_CS model can reflect the creep law of the clayey soil in this area.

## Conclusions

This paper analyzes the characteristics of groundwater level change and the physical state and stress state of deep clayey soil in the land subsidence area of Cangzhou, and investigates the creep behavior of deep clayey soil by using compression tests. Combined with the test data and theoretical analysis, the study proposes a creep model of clayey soil and draws the following conclusions.

1. The test results show that the total strain under stepwise loading consists of three parts: instantaneous strain, primary consolidation strain and creep strain. According to the Boltzmann superposition principle, the creep curves and isochronous stress–strain curves under stepwise loading are obtained, and both of them have obvious nonlinear characteristics, and the creep curves as a whole show a steady state development trend.
2. Based on the Merchant model, the NCE_CS nonlinear creep model was established by replacing the elastic element in the model with a power function element and improving the Kelvin body of the Merchant model so that the onset time of creep can be considered. The NCE_CS model introduces the onset time of the creep stage, which makes the division of the primary consolidation and creep stages clear and effectively reflects the nonlinear creep characteristics of clayey soils.
3. The NCE_CS model has five parameters, among which the modulus *E*_*se*_ can be obtained by one-dimensional compression test, and the remaining four parameters *a*, *b*, *c*, and *η* can be calculated by genetic algorithm. The calculated results of the NCE_CS model were compared with those of the test data and four classical creep models. The results show that, compared with the classical creep models, the creep curves and isochronous stress–strain curves calculated by the NCE_CS model are in better agreement with the test data, indicating that the NCE_CS model can better reflect the creep deformation characteristics of clayey soils.
4. To further verify the applicability of the NCE_CS model, the creep curves of the clayey soils in another subsidence area, located in Renqiu City in northwest of Cangzhou, were obtained by using this model, with most of the *SR* values ranging from 0.9 to 1.2, indicating that the model calculations are highly accurate. Once again, it shows that the NCE_CS model can effectively reflect the creep properties of clayey soils in Cangzhou area.

## Data Availability

The original contributions presented in the study are included in the article/Supplementary Material, further inquiries can be directed to the corresponding author.
